# The Effectiveness of Modular Transdiagnostic Cognitive Behavioral Therapy Versus Treatment as Usual for Youths Displaying School Attendance Problems: A Randomized Controlled Trial

**DOI:** 10.1007/s10802-024-01196-8

**Published:** 2024-05-13

**Authors:** Daniel B. Johnsen, Johanne J. Lomholt, David Heyne, Morten B. Jensen, Pia Jeppesen, Wendy K. Silverman, Mikael Thastum

**Affiliations:** 1 Department of Public Health, University of Copenhagen, Copenhagen, Denmark; 2 Department of Psychology and Behavioral Sciences, Aarhus University, Aarhus, Denmark; 3 TrygFonden’s Centre for Child Research, Aarhus University, Aarhus, Denmark; 4 School of Psychology, Deakin University, Melbourne, Australia; 5 Department of Economics and Business Economics, Aarhus University, Aarhus, Denmark; 6 Department of Child and Adolescent Psychiatry, Copenhagen University Hospital – Psychiatry Region Zealand, Roskilde, Denmark; 7 Department of Clinical Medicine, Faculty of Health and Medical Sciences, University of Copenhagen, Roskilde, Denmark; 8 Child Study Center, Yale University School of Medicine, New Haven, USA

**Keywords:** School attendance problems, Cognitive behavioral therapy, Modular, Anxiety, Depression, Behavioral problems

## Abstract

School attendance problems (SAPs) are associated with negative short- and long-term outcomes. Despite high prevalence of SAPs, there is a shortage of evidence-based interventions. Existing approaches often target either school refusal or truancy, leaving a gap in effective interventions addressing both types of SAPs. This randomized controlled trial (RCT) assessed the effectiveness of Back2School (B2S), a modular transdiagnostic cognitive behavioral therapy (CBT) for SAPs, compared to treatment as usual (TAU). Outcomes included youths’ school attendance and mental health. A group (B2S, TAU) × time (Pre, Post, 3-Month Follow-Up) design involving 152 youths (B2S; *n* = 74, TAU; *n* = 78) with SAPs (i.e., ≥ 10% absence in the past three months), aged 6–16 years (*M* = 12.2, *SD* = 2.2, males = 60%) were used. B2S comprised three months of CBT with youths, parents, and school involvement, while TAU comprised public and/or private intervention services. On average, youths in B2S received 15.0, (*SD* = 3.9) hours of intervention, while those in TAU received 13.4, (*SD* = 21.6). Intervention effects were investigated using mixed linear models. Both B2S and TAU exhibited significant within-group improvements in school attendance, with no significant differences between them. However, the B2S group significantly outperformed TAU in reducing youths’ emotional problems, conduct problems, problems with peers, the overall impact of problems, and increasing youths self-efficacy for attending school and parent self-efficacy for dealing with a SAP. This RCT represents the first evaluation of a modular transdiagnostic CBT for youths displaying SAPs, showing significant mental health and self-efficacy benefits.  (Clinical trial registration: ClinicalTrials.gov: NCT03459677).

**Supplementary Information:**

The online version contains supplementary material available at 10.1007/s10802-024-01196-8.

## Background

School attendance problems (SAPs) refers to difficulty attending school or absence from school that is problematic because of its frequency and/or duration (Heyne et al., 2019). SAPs are associated with negative outcomes for youths such as poor academic achievement (Gottfried, 2014), school dropout (Schoeneberger, 2012), and later unemployment (Attwood & Croll, 2015). Studies further show that most youths displaying SAPs experience a range of mental health problems including depression, anxiety, and behavioral problems (Askeland et al., 2015; Egger et al., 2003; Finning et al., 2019; Vaughn et al., 2013). In various countries, missing 10% or more of school is regarded as the threshold for the presence of a SAP, and prevalence rates of youths crossing this threshold range from 11 to 25% in Denmark, Australia, the UK, and the USA (Australian Curriculum Assessment and Reporting Authority, 2023; Danish National Agency for IT and Learning, 2019; Department for Education, 2019; U.S. Department of Education, 2019). Rates of SAPs typically increase with age and are more prevalent among older youths (Gubbels et al., 2019; Heyne, 2022). Because SAPs are prevalent and associated with negative short- and long-term outcomes, there is need for effective, scalable, and accessible interventions.

Previous research on SAPs has often focused on one of two types, namely *truancy* or *school refusal.* Truancy involves unauthorized non-attendance or ‘skipping’ school, while school refusal refers to non-attendance associated with emotional distress (Heyne et al., 2019). This distinction between truancy and school refusal has impacted the development of SAP interventions, inasmuch as interventions are often designed to address one of these two types (Heyne et al., 2015). Truancy interventions primarily aim to increase school attendance by involving various stakeholders, including the young person (e.g., through mentoring), parents/families (e.g., via parent training), or the school/community (e.g., by fostering school bonding) (DeSocio et al., 2007; Franklin et al., 2007; Mazerolle et al., 2017). School refusal interventions often focus on improving school attendance using cognitive behavioral therapy (CBT), incorporating graded exposure and cognitive restructuring techniques to reduce mental health problems related to symptoms of anxiety and/or depression (Heyne et al., 2011; Heyne & Sauter, 2013). Similar to truancy interventions, school refusal interventions engage not only the youths but also parents and school personnel [e.g., (Heyne et al., 2002; Melvin et al., 2017)]. Promisingly, meta-analyses of truancy interventions (*g* = 0.46; (Maynard et al., 2013)) and school refusal interventions (*g* = 0.54; (Maynard et al., 2018)) have demonstrated overall positive and significant effects on attendance.

The fact that truancy and school refusal are commonly perceived as different types of SAPs does not mean that they are mutually exclusive. Both may be associated with mental health problems and disorders, including anxiety, depression, behavioral problems, and other disorders such as autism spectrum disorder and attention deficit/hyperactivity disorder (Egger et al., 2003; Heyne et al., 2019). Therefore, adopting a modular transdiagnostic approach that address different types of SAPs, as well as different types of co-occurring mental health problems appears to be a viable and innovative path forward on conceptual, empirical, and clinical levels (Kearney & Graczyk, 2020). Although evidence-based interventions are available for several mental health problems, many youths with emotional or behavioral problems do not receive them (Costello et al., 2014). Studies show that transdiagnostic approaches have a potential for high scalability, that could make evidence-based practice more readily available (Jeppesen et al., 2021; Weisz et al., 2017).

Evidence supports the effectiveness of modular CBT interventions in addressing anxiety, depression, and behavioral problems (Ginsburg et al., 2020; Jeppesen et al., 2021; Weisz et al., 2012). In a cluster randomized clinical trial, Weisz and colleagues (2012) found that youths receiving modular CBT had fewer diagnoses post-treatment compared to those receiving usual care. However, an individual-level randomized clinical trial of the same modular intervention did not demonstrate its superiority over usual care (Weisz et al., 2020). In a recent individual-level randomized clinical trial, Jeppesen and colleagues (2021) found that a modular transdiagnostic CBT, Mind My Mind (MMM), outperformed management as usual in a community setting across various clinically relevant domains for youths experiencing emotional and behavioral problems. Ginsburg and colleagues (2020) also showed that modular CBT was effective in reducing symptoms of anxiety, in a school-based outpatient setting. These findings collectively suggest that modular transdiagnostic CBT holds promise as a viable approach for addressing the complex needs of youths with multiple co-occurring problems, including SAPs.

No prior study has used a modular transdiagnostic CBT approach specifically tailored to address SAPs. Several studies, however, have used CBT to increase school attendance among youths diagnosed with mental health problems (Hannan et al., 2019; Heyne et al., 2002; Reissner et al., 2015; Walter et al., 2010). In a comprehensive examination, Reissner and colleagues (2015) evaluated the impact of a protocol-guided multimodal treatment (MMT) for youths with confirmed mental disorders and SAPs, comparing it to treatment as usual (TAU). The MMT included modules of CBT, family counseling, school-related counseling, and a psychoeducational exercise program. Interestingly, both arms witnessed significant improvements in attendance, but no difference emerged between the two. Although there is an association between SAPs and mental health problems, youths can also develop SAPs without having a mental health diagnosis. Thus, it becomes imperative to develop programs capable of addressing a broad spectrum of SAPs, ensuring that youths with SAPs, with and without coinciding mental health diagnosis, receive the help they require.

We developed the Back2School (B2S) program, a manualized outpatient CBT intervention for youths displaying SAPs. It was designed to be used alongside the modular MMM-CBT manual. The MMM manual comprises evidence-based CBT methods and techniques organized into disorder-specific modules, targeting both subclinical and clinical levels of anxiety, depression, behavioral disturbances, and trauma-related problems. The B2S manual refers to relevant material from the MMM manual and aims to increase school attendance by using CBT procedures from that manual, including parent management, contingency management, incentives for attendance, cognitive restructuring, and exposure-based practices.

Research consistently indicates a significant increase in self-efficacy for managing situations related to school attendance following CBT interventions for school refusal (Heyne, 2022; Heyne et al., 2011; King et al., 1998). There is also research indicating that truancy interventions can improve school engagement and self-efficacy (DeSocio et al., 2007; Mann et al., 2015). Moreover, it is suggested that intervention that increases self-efficacy may yield reductions in mental health problems such as anxiety and facilitate reengagement with schooling (Heyne et al., 2015). At the core of the B2S intervention lies the strategic use of graded exposure, whereby youths have success experiences as a result of successively manageable exposure-based tasks, helping bolster their self-efficacy.

Our preliminary feasibility trial of the B2S intervention (i.e., B2S implemented concurrently with MMM-CBT) yielded promising results, demonstrating its feasibility and acceptability in addressing SAPs. This trial revealed significant improvements in both youths’ school attendance and mental health, coupled with high levels of treatment satisfaction (Lomholt et al., 2020). Building upon these insights, we used the feasibility trial findings to refine the B2S manual and strengthen the current study protocol. Key modifications to the B2S manual were implemented to improve collaboration with schools, involving revisions in the planning and implementation of school meetings. We identified possible limitations regarding the school attendance data provided by the municipality, and therefore we added parent-reported school attendance data (e.g., hours of attendance) as one of our primary outcomes (Lomholt et al., 2020; Thastum et al., 2019).

The primary aim of the current study was to rigorously evaluate the effectiveness of B2S, comparing it with an active comparator control arm that received TAU. We formulated three primary hypotheses. First, we hypothesized that the B2S intervention would outperform TAU in increasing both the number of hours and days of school attendance. Second, we hypothesized that B2S would outperform TAU in reducing symptoms associated with emotional, behavioral, and social difficulties, ultimately diminishing their interference with daily life. Our final hypothesis was that the B2S intervention would lead to a significantly greater increase in youths’ self-efficacy for managing school situations and parents’ self-efficacy for managing a SAP when compared to TAU. Additionally, we conducted a comparative analysis of participant rating of treatment satisfaction between the two groups.

## Methods

### Design

**Fig. 1:**
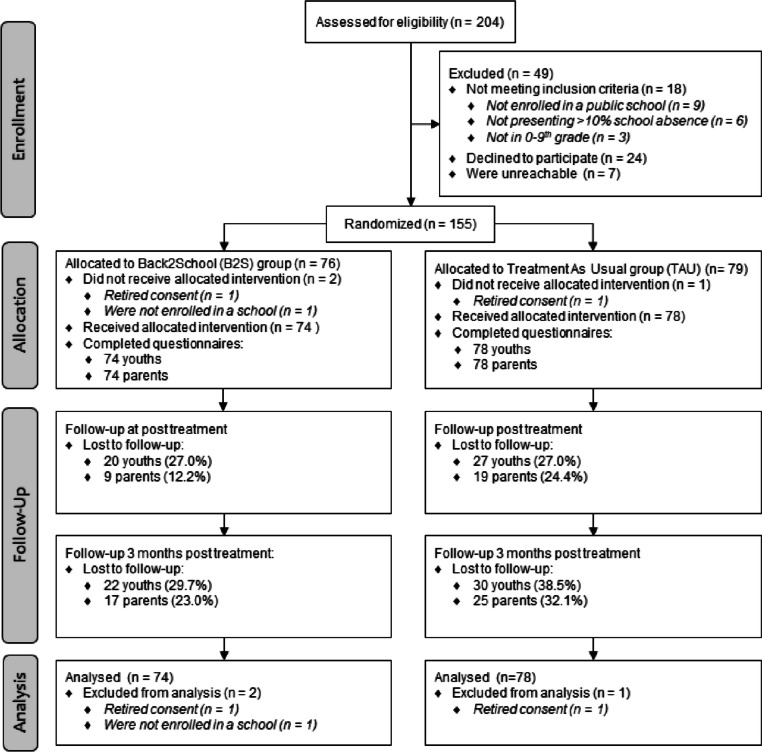
Consort diagram

The study was a pragmatic, single site, superiority RCT, employing a mixed within-between-subjects design to evaluate the effectiveness of the B2S intervention, compared with TAU. Participants in both arms were assessed on primary and secondary outcomes prior to randomization and allocation to arm (Pre), immediately after the intervention period, which we expected to be approximately after 4 months including delays and extra sessions for the B2S group and 4 months after the randomization for the TAU group (Post), and at three months following treatment (3FU). The overall study design is illustrated in Fig. 1. Our initial study design (Thastum et al., 2019) included a 12-month follow-up (12FU). Planned data collection at 12FU overlapped with the national restrictions and closure of Danish schools due to the COVID-19 pandemic. In this period the Danish government closed all schools and implemented changes in the policies for registering school attendance, whereby schools were no longer obligated to register school attendance. Therefore, valid data from the 12FU period, is not available.

### Participants

Two-hundred-and-four youths were assessed for eligibility. Forty-nine youths were excluded for various reasons (see consort diagram, Fig. 1). One-hundred-fifty-five youths met inclusion criteria and were randomly assigned to B2S (*n* = 76) or TAU (*n* = 79). However, two participants withdrew their consent to participate in the trial and requested their data to be deleted and they were therefore excluded from the intention-to-treat analyses. Due to incorrect information from the parents, one participant was wrongly included and randomized, although the inclusion criteria of being enrolled in a public school was not fulfilled. Therefore, this case was excluded from the intention-to-treat analyses as well (excluded participants: B2S: *n* = 2, TAU: *n* = 1). The number of available participants for each intervention arm is shown in Fig. 1.

Participants in the intention-to-treat (ITT) analyses of the RCT included 152 youths displaying SAPs (*n* = 92 males) between 6 and 16 years of age (*M* = 12.2 years, SD = 2.2), and their parents. The majority of the sample (*n* = 119) consisted of youths in secondary education (e.g. 11–16 years). Participants were recruited between August 1st, 2017, and March 31st, 2019, whereby families self-referred for treatment for a SAP. Families were required to make initial contact to participate in the study, encouraged by promotional/recruitment material for the project. Families could be informed about and directed to the study by health or education professionals, but could not be formally referred. Prior to and throughout the recruitment period, the municipality implemented extensive information and media campaigns aimed at families and professionals. Participants could contact project coordinators with questions via telephone or e-mail. Eligibility for participation was ascertained through a brief online screening accessed through the project web page completed by a parent or another legal guardian/caretaker, based on the following inclusion criteria: (a) young person enrolled in a public school within the region of central Denmark; (b) young person aged 6–16 years and in 0–9th grade (excluding the second semester of ninth grade); (c) parent reported more than 10% school absence on behalf of their child during the previous three months of school; (d) the young person and at least one parent understood and spoke Danish sufficiently well to participate in treatment and complete questionnaires; (e) at least one parent was motivated to work on increasing the youth’s school attendance; (f) young person and parent(s) were committed to participate in assessment and intervention procedures, and willing to accept random assignment to intervention; and (g) the holders of the parental rights gave written informed consent for participation, and likewise for the young person of age 15–16. Regarding criterion (c), parents were asked to indicate the amount of school their child missed during the last three months, using the following six categories: ‘Less than 10% (less than six absent days)’, ‘10–20% (approximately 6–12 absent days)’, ‘20–30% (approximately 12–18 absent days)’, ‘30–50% (approximately 18–30 absent days)’, ‘ > 50% (more than 30 absent days)’, and ‘100% (the child has not attended school in the last three months)’.

### Interventions

#### Back2School

 Is a modular CBT program aimed at helping youths displaying SAPs to increase their school attendance by involving the youths, their parents, and school personnel in the intervention. It consists of an assessment interview with the young person and their parent(s), development of a case formulation, 8 sessions of CBT with the young person and the parents together, 2 sessions with the parents, and 3 school meetings, all conducted within a three-month period. The B2S treatment period was expected to take approximately 4 months, when accounting for including delays due to school holidays. Approximately three months after the 10th session there is a booster session with the young person and the parents together, and a booster meeting with school personnel and the parents. For a detailed overview of the intervention, see Table 1.

**Table 1:** Overview of the Back2School program

**Session number**	**Duration (hours)**	**Participants**	**Session content**
S-0	1.5	T, Y, P	Therapists conduct a structured assessment interview with the family, providing psychoeducation and SMART goals handouts as homework for the next session.
Clinical conference	1	T	The therapists are discussing the case formulation, choice of treatment modules, and treatment goals with a colleague.
S-1	1	T, Y, P	The family is presented with and discusses the case formulation. The session includes psychoeducation about school absence and the development of SMART goals.
S-2	1	T, P	Therapists assist parents in addressing questions or problems related to school placement, child's somatic symptoms, and parental motivation. This includes planning better home routines and addressing potential sleep problems.
S-3	1	T, Y, P	Planning the date for returning to school and planning the first day back in school. Creating a gradual exposure plan for returning to school.
S-4	1	T, Y, P	Psychoeducation regarding the youth’s primary problem related to school absence (e.g., anxiety, depression, or behavioral problems) from the MMM modules. Continued work with the gradual exposure plan for returning to school.
S-5	1	T, Y, P	Application of CBT methods addressing the youth’s primary problem related to school absence (e.g., exposure, behavioral activation and/or cognitive restructuring) from the MMM modules. Continued work with the gradual exposure plan for returning to school. Work with parents involves setting boundaries.
S-6	1	T, P	Working with parent behavior. Identifying and reducing factors at home that maintain school absence.
S-7	1	T, Y, P	Continued work towards returning to school, and revision of gradual exposure plan. Work with parents involves how they can support the youth in exposure exercises and returning to school. Includes problem solving.
S-8	1	T, Y, P	Open session tailored to the needs of the youth and parents. Continued work with CBT methods from the MMM modules.
S-9	1	T, Y, P	Open session tailored to the needs of the youth and parents. Continued work with CBT methods from the MMM modules.
S-10	1	T, Y, P	Concluding the B2S program. Focusing on maintaining progress.
Booster	1	T, Y, P	Focusing on maintaining progress. Problem solving regarding relevant problems. Discuss further assistance if needed.
SM 1	1	T, P, S, Y	Presenting and discussing the case formulation with the school. Planning the school’s role in the youth’s return to school. Informing the school about the B2S and CBT approach.
SM 2	1	T, P, S, Y	Following up on the youth’s progress in the school setting. Discussing potential academic difficulties, problems regarding bullying or other problems.
SM 3	1	T, P, S, Y	Planning how the school can continue to help and support the youth. Discussing relapse prevention.
SM 4	1	T, P, S, Y	Planning how the school can continue to help and support the youth. Discussing relapse prevent.

*S *Session, *SM *School meeting, *Y *Youth, *P *Parent, *T *Therapist, *S *School officials, *MMM *Mind My Mind, *B2S *Back2School, *CBT *Cognitive Behavioral Therapy

The B2S intervention (Thastum et al., 2019) was specifically developed to treat SAPs, drawing on components of the @School intervention (Heyne et al., 2014; Heyne & Sauter, 2013) and the When Children Refuse School intervention (Kearney & Albano, 2007). As previously mentioned families self-referred for treatment for SAPs, and it was a mandatory goal of the treatment, to be agreed upon by the family, to work on increasing the youth´s school attendance. The B2S intervention was implemented in conjunction with the modular MMM manual, which includes evidence-based CBT methods organized into disorder-specific modules to target subclinical or clinical levels of anxiety, depression, and behavioral disturbance (Jeppesen et al., 2021). The MMM manual thus supplements the B2S manual, and the B2S manual refers to relevant material from the MMM manual when relevant. Intervention strategies are determined through a descriptive functional analysis together with a case formulation approach to planning CBT for attendance problems. The functional approach involves identifying the motivational function underlying the youth´s school attendance issues. School absenteeism motivated by positive reinforcement suggests CBT procedures such as parent management, contingency management and contracting to minimize incentives for SA and boost incentives for attendance. School absenteeism motivated by negative reinforcement suggests CBT procedures such as cognitive restructuring, and exposure-based practice to reduce the anxious or depressive physical sensations and thoughts.

Initially the families attend a 1.5-h structured clinical interview, held by the appointed therapists. The interview is designed to get an understanding of the youth’s SAP, development, family and social situation, and functioning in daily life. The interview also includes a brief, semi-structured psychopathological interview developed for the study with the youth and parents together. Based on the qualitative and quantitative information derived from the interview and the pre-intervention assessment battery, a case formulation is developed by the therapists. At a clinical case conference, the case formulation is discussed with a clinical psychologist, a primary problem is defined (e.g., anxiety, depression, or behavioral problem), and a preliminary treatment plan is constructed. If relevant this might include treatment for anxiety, depression and/or behavioral problems. At the first session, the case formulation is discussed with the family with the aim of achieving a common understanding of the maintaining factors for the SAPs and developing a treatment plan. Additionally, during the first school meeting, the case formulation is also discussed with the school staff, with the aim of fostering a shared understanding of the SAPs between the family and the school staff (Thastum et al., 2019). Older youths were in some cases invited to participate in the school meetings, when the therapist found it relevant and appropriate.

Participating families in the B2S group completed a mean of 10.0 (SD = 2.45, range 0–11) of the therapy sessions and booster session. Fifty-eight (*n* = 58/74, 78%) families completed all therapy sessions and the booster session. On average, families completed a mean of 3.15 (SD = 1.08, range 0–4) of the four school meetings (including the booster school meeting). Thirty-seven (*n* = 37/74, 50%) families completed all four school meetings. If the B2S psychologists deemed it necessary, and after consultation with the research leader, families were offered extra meetings or extra therapy sessions (e.g., to coordinate future interventions with other professionals, or to ensure the wellbeing of the families) after the B2S intervention was completed. Eighteen families (*n* = 18/74, 24.3%) received at least one extra meeting or therapy session, with a mean of 1.7 (SD = 1.2, range 0–5) extra meetings or therapy sessions. The mean number of sessions received in the B2S intervention (including school meetings) was 14.6 (SD = 2.3, range 1–18), and the mean number of hours of intervention received was 15.0 (SD = 3.9, range 1.5–20.6) for the families in the B2S group. From intervention allocation to the last completed therapy session (excluding the booster session), the average intervention time was 4.2 months.

Following the clinical assessment, the youth’s primary problem (e.g., anxiety, depression, or behavioral problem) was identified and modules pertaining to their primary problem were included in the individualized treatment plan. Information regarding the utilized treatment modules was available for 61 (82.5%) of the families. Of these, 41 (67%) families began with anxiety modules, 9 (15%) began with depression modules, and 11 (18%) began with behavioral problems modules. Eleven families (15%) transitioned to another type of modules during the treatment: 1 family started with behavior modules and supplemented with depression modules, 2 families started with depression and shifted to anxiety modules, 1 family started with depression modules and supplemented with behavioral problems modules, and 7 families started with anxiety modules and shifted to behavioral problems modules.

Fidelity checks for the B2S intervention were conducted by three raters, with previous training and experience in conducting fidelity checks in the MMM RCT (Jeppesen et al., 2021). They also received an additional two-day introduction to the B2S manual. The raters evaluated the psychologists' competence conducting CBT and their adherence to the intervention manuals (i.e., B2S and MMM) by rating video recorded sessions using the *Competence and Adherence Scale for Cognitive Behavioral Therapy for Transdiagnostic Modular based Manuals* (CAS-CBT-TMM; (Bjaastad et al., 2015). In total, 25 (33.8%) of the 74 participants in the B2S group, were randomly selected for the fidelity checks. For each participant, three video recorded sessions were randomly selected; one session from early treatment (i.e., session 1–3), one session from mid-treatment (i.e., session 4–6), one session from late treatment (i.e., session 7–10). In total, 75 sessions were randomly selected and used in the assessment of treatment fidelity. Each rater assessed 25 randomly selected sessions, and five sessions randomly selected from another rater, to be used as a measure of inter-rater reliability (30 sessions in total). Global *competence* was rated on a 7-point scale (0 = *Poor skills*, 6 = *Excellent skills*), yielding an acceptable level of psychologists’ average competence (*M* = 3.24, SD = 1.30) (Bjaastad et al., 2015). Global *adherence* was rated on a 7-point scale (0 = *None,* 6 = *Thorough*), yielding an acceptable level of psychologists’ average adherence (*M* = 3.44, SD = 1.29) (Bjaastad et al., 2015). Inter-rater reliability checks yielded good agreement (based on interclass correlations [ICC]) for ratings of adherence (ICC = 0.633) and competence (ICC = 0.620) (Cicchetti, 1994).

#### Treatment as usual

In accordance with Danish law, schools are responsible for helping youths displaying SAPs (Danish Ministry of Children and Education, 2019). In effect, when school personnel initiate and provide help for a young person displaying a SAP, using available resources within the school system, this is TAU. Families randomly allocated to TAU were not restricted in seeking interventions or assistance outside the school, and thus TAU could include treatment from private psychologists, psychiatric hospitals, or physicians. Information about the interventions received in the TAU group was obtained using a *semi-structured telephone interview* with the parents or legal guardian/caretaker in the TAU group. The interviews gathered information regarding the type(s) of intervention families participated in, who provided the intervention, number of meetings, and duration of the intervention during a treatment period of 4 months. Sixty of the 78 families participating in TAU (77%) completed the semi-structured interview following intervention; the remaining families were either unreachable (*n* = 12; 15%) or declined to participate in the interview (*n* = 6; 8%). Information from the interviews indicated that 59 of the 60 families participating in TAU (98%) received intervention or help provided through public services, and 19 through private services (32%). Participants (n = 60) in the TAU group reported that they received on average 13.4 h of intervention (SD = 21.6, range 1–116) between Pre and Post assessment (see Online Resource 1), however the variance was much higher in the TAU group with some cases who received more than 100 h of intervention.

### Measures

A synopsis of the measures is presented below; see the study protocol for details of these measures (Thastum et al., 2019).

#### Primary outcome measures

##### Hours of school attendance

was assessed using parent retrospective reports of their child’s hourly school attendance during the ten school days immediately preceding Pre, Post, and 3FU. A percentage of hours of school attendance was calculated for the previous ten school days.

##### Days of school attendance

was assessed using attendance data provided by the local municipalities’ school attendance registries (i.e., registered as ‘in attendance’ or ‘absent’). A percentage of days of school attendance was calculated for the days of attendance in the last ten school days.

#### Secondary outcome measures

##### Youths symptoms of emotional, behavioral, and social difficulties, and the interference caused by these difficulties,

were measured using the extended version of the Strength and Difficulties Questionnaire, as reported by youths aged 11 years and older (SDQ) and all parents (SDQ-P) (Goodman, 1997, 1999). The SDQ is a brief behavioral screening questionnaire and consists of 25 items rated on a 3-point scale. The items are divided into five 5-item subscales that generate a score for emotional symptoms, conduct problems, hyperactivity/inattention, peer relationship problems, and prosocial behavior. The total difficulties scale sums up the difficulties across the four problem areas (not including lack of prosocial behavior). The extended version of the SDQ includes questions regarding youths’ distress and interference of problems with home life, friendships, classroom learning, and leisure activities, each scored on a 4-point scale. The impact scale sums up the distress and interference of problems, counting only the moderate and severe levels. The internal consistency for the SDQ total scale in the current sample was α = 0.81 for youths and α = 0.76 for parents.

##### Youths self-efficacy

for handling situations related to school attendance was measured via the Self-Efficacy Questionnaire for School Situations (SEQ-SS) (Heyne et al., 1998). The SEQ-SS consists of 12 items and 2 subscales: academic/social stress and separation/discipline stress. Each item measures self-efficacy expectations related to different school situations on a 5-point scale. The total score is derived from summing the items together. The internal consistency for the SEQ-SS total scale in the current sample was α = 0.82.

##### Parental self-efficacy

for responding to their child’s SAP was measured via the Self-efficacy Questionnaire for Responding to School Attendance Problems (SEQ-RSAP) (Heyne et al., 2016). The SEQ-RSAP consists of 13 items assessing parents’ self-efficacy for dealing calmly and constructively with the youth’s difficulty attending school, rated on a 4-point scale. The total score is derived from summing the items together. The internal consistency for the SEQ-RSAP scale in the current sample was α = 0.83.

#### Additional measures

Parents provided developmental and demographic data at Pre (e.g., developmental delays for the young person, living situation).

##### Possible adverse events and overall treatment satisfaction 

were assessed by the parents at Post using the Experience of Treatment Satisfaction Questionnaire (ESQ) developed for the current study. Parents were asked to rate ten statements on a three-point Likert scale (1 = not true; 2 = partly true; 3 = true). Exemplary items are: ‘The treatment helped me/my child’, ‘We feel better in the family now compared to before the treatment’, ‘If a friend needed this type of help, I would recommend him/her to contact the clinic’, and ‘The treatment made me feel worse’ (scores reversed for negative statements).

## Procedure

Participants meeting inclusion criteria received a complete description of the study, gave written informed consent, and were subsequently randomized to one of the two intervention arms: B2S or TAU. Randomization was performed using a computer-generated random digit procedure with two possibilities (B2S and TAU). The primary outcome, increase in school attendance, may be affected by youths age and by the amount of school non-attendance prior to intervention (Heyne et al., 2015). Therefore, to ensure balanced arms, the randomization was stratified on the presence of age (first to fourth grade [younger] or fifth to ninth grade [older]) and amount of school non-attendance (< 50% [low] or > 50% [high]). To maintain similar intervention arms sizes, the randomization was conducted using permuted block randomization. The block size and computer-generated allocation lists were concealed from the research team overseeing the RCT study, throughout the recruitment period. Following randomization to either B2S or TAU, all participants received written and verbal information regarding intervention allocation from the research team. To increase retention of participants in the TAU group, the families were offered a gift card (value 200 DKK/26 EUR) after completing the Post and/or FU assessment. After randomization participants in the B2S group were notified of the time and place of the start of the intervention, and participants in the TAU group were urged to contact their school to get help for the SAP. The participant’s school and the school leader were also notified of the outcome of randomization. The last follow-up assessment (3FU) was in December 2019, for both interventions.

The B2S intervention was delivered by 8 psychologists, and 37 clinical psychology master students serving as co-therapists. The psychologists worked as school psychologists in Aarhus Municipality and were hired part-time to deliver the B2S intervention. Prior to the current study, all psychologists and co-therapists had limited knowledge and experience with CBT and were regarded as novice CBT therapists. Psychologists and co-therapists received a 6-day training course prior to the trial with an introduction to clinical assessment, case formulation, and the B2S and MMM manuals. They also received four 1-day booster training sessions during the study period. In total, psychologists and co-therapists received between 60 and 80 h of training, comparable to previous studies using modular trans-diagnostic CBT (Jeppesen et al., 2021; Weisz et al., 2012). All psychologists and co-therapists received weekly face-to-face group case supervision by specialists in clinical child psychology, for the duration of the trial (i.e., the two years in which interventions were delivered).

## Statistical Analysis

The study was powered to provide 80% power at the 5% (two-tailed) significance level to detect a standardized effect size within the range of 0.46–0.54 difference in the primary outcome (change in school attendance), following Maynard and colleagues (Maynard et al., 2013, 2018). To account for the expected attrition rate of 10% (Heyne et al., 2002; King et al., 1998; Last et al., 1998; Wu et al., 2013), the required sample size of 70 participations per intervention group was raised to 77 per group. Mixed linear models (MLMs) were used to determine the time × group interaction effects and the effects of intervention groups over time. MLMs allow for handling of the time variable as a time-varying covariate. Thus, the time variable was calculated from each participant's day of assessment for all time-points. MLMs accommodate missing values in the dependent variable as an integral part of the analysis and do not compromise statistical power. All MLMs were estimated using the maximum likelihood method and based on the intention-to-treat sample. All models included a random intercept, and the slope was specified as random if improving the model fit (evaluated by a significant change in the -2 log-likelihood (-2LL) fit statistics (Heck et al., 2012). To explore the robustness of the primary and secondary outcomes, we estimated unadjusted mean changes and differences in mean changes with corresponding 95% CIs for significant time × group interactions. The mean score from the ESQ measure was compared between intervention groups using an independent *t*-test. Effect sizes were calculated for all outcome measures, expressed as Cohen’s *d*, with 0.2, 0.5, and 0.8 considered small, medium, and large effect sizes, respectively (Cohen, 1988). Cohen’s *d* was derived from the *F* test calculated as *d* = 2 × √ (F / df). IBM SPSS Statistics, v.26 was used for all analyses.

## Results

### Baseline Comparisons

The participant baseline demographics are presented in Table 2. The attendance records provided by the municipalities yielded information for 124 youths about the percentage of school attendance for each month during the academic year prior to study inclusion. Of these 124, 64 had been randomly allocated to the B2S group and 60 to the TAU group. The average school attendance among youths in each group had decreased across the 10 months prior to study inclusion, from 81% attendance to 56% for youths in the B2S group, and from 84 to 53% for youths in the TAU group (Online Resource 2).

**Table 2:** Baseline clinical and sociodemographic characteristics for both intervention groups

	B2S (*n* = 74)	TAU (*n* = 78)
Age, mean (SD)	12.3 (2.1)	12.0 (2.2)
Gender, males, no. (%)	46 (62.2)	46 (59.0)
Level of non-attendance previous 3 months:		
High (> 50%), no. (%)	38 (51.4)	47 (60.3)
Low (< 50%), no. (%)	36 (48.6)	31 (39.7)
Age group:		
6–10 years, no. (%)	13 (17.6)	20 (25.6)
11–16 years, no. (%)	61 (82.4)	58 (74.4)
Physical illness (e.g., asthma, allergies), no. (%)	15 (20.3)	19 (24.4)
Developmental delays^a^, no. (%)	14 (18.9)	26 (33.3)
Developmental or psychological disorder:		
Anxiety disorder, no. (%)	10 (13.5)	8 (10.3)
Depressive disorder, no. (%)	8 (10.8)	0 (0.0)
Attention Deficit Hyperactivity Disorder, no. (%)	8 (10.8)	8 (10.3)
Autism Spectrum Disorder, no. (%)	6 (8.1)	6 (7.7)
Learning disability, no. (%)	7 (9.5)	3 (3.8)
Intellectual disability, no. (%)	1 (1.4)	2 (2.6)
Conduct disorder, no. (%)	0 (0)	1 (1.3)
Other^b^, no. (%)	1 (1.4)	3 (3.8)
Comorbidity, ≥ 2 disorders, no. (%)	13 (17.6)	10 (12.8)
Any disorder, no. (%)	22 (29.7)	18 (23.1)
Living arrangement:		
Both parents, no. (%)	41 (55.4)	43 (55.1)
Single parent, no. (%)	22 (29.7)	19 (24.4)
Other/reconstituted family, no. (%)	11 (14.9)	16 (20.5)
Number of siblings in the household:		
Only index child, no. (%)	20 (27.0)	14 (17.9)
1, no. (%)	27 (36.5)	38 (48.7)
2, no. (%)	23 (31.1)	23 (29.5)
≥ 3, no. (%)	4 (5.4)	3 (3.8)

*B2S *Back2School, *TAU *Treatment As Usual, *SAP *School Attendance Problems, *SCAS *Spence Child’s Anxiety Scale, *MFQ *Mood and Feelings Questionnaire, *SDQ *Strength and Difficulties Questionnaire

^a^Language (B2S: n = 6, TAU: n = 11), Motor skill (B2S: n = 4, TAU: n = 4), Social interaction (B2S: n = 2, TAU: n = 6), Learning (B2S: n = 1, TAU: n = 1)

^b^Functional Somatic Symptoms (B2S: n = 1), Trauma (TAU: n = 2), Tourette (TAU: n = 1)

At baseline there was a discrepancy between the attendance reported in the school attendance registries and what parents reported regarding their children's attendance the last 10 days. Twenty-seven youths (17.7%) had 100% attendance according to the school attendance registries. However, only two youths (1.4%) had 100% attendance according to what their parents reported for hours of school.

### Primary Outcomes

**Fig. 2:**
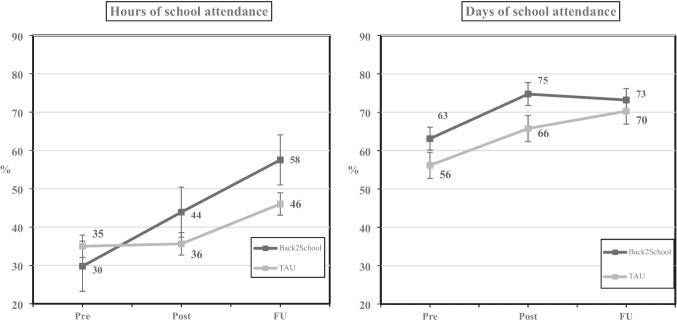
Mean school attendance (%) at Pre, Post and 3-month follow up

Outcomes related to school attendance are presented in Table 3 and Fig. 2. For *hours of school attendance* as reported by parents, there was no significant time × group interaction effect (*F* = 2.61, *p* = 0.11,* d* = 0.26). There was a significant increase in *hours of school attendance* from Pre to FU for youths in the B2S group of 27.7% (*F* = 26.96, *p* =  < 0.01,* d* = 1.14) and for youths in the TAU group of 11.0% (*F* = 9.41, *p* =  < 0.01,* d* = 0.75). For *days of school attendance* as reported in local municipalities’ school attendance registries, no significant time × group interaction effect was found (*F* = 0.14, *p* = 0.71,* d* = 0.04). There was a significant increase in *days of school attendance* from Pre to FU for youths in the B2S group of 10.1% (*F* = 9.85, *p* =  < 0.01,* d* = 0.51) and youths in the TAU group of 14.1% (*F* = 12.44, *p* =  < 0.01,* d* = 0.58).

**Table 3:** Means, standard deviations and main effects for primary, secondary, and additional outcomes for the B2S (n = 74) and TAU (n = 78) group

	Group	Pre	Post	3FU	Time	Time-by-group
		*M* (SD) [valid n]	*M* (SD) [valid n]	*M* (SD) [valid n]	*F, P* (Cohen’s *d*)	*F, P* (Cohen’s *d*)
School Attendance – Hours, Two weeks (%)	B2S	29.80 (27.24) [74]	43.91 (33.10) [65]	57.53 (37.31) [54]	26.96, < 0.001 (1.14)	2.61, 0.108 (0.26)
	TAU	35.01 (29.45) [68]	35.64 (35.16) [56]	46.03 (37.18) [53]	9.41, 0.003 (0.75)	
School Attendance – Days, Two weeks (%)	B2S	63.11 (32.94) [74]	74.73 (27.76) [74]	73.19 (31.26) [72]	9.85, 0.002 (0.51)	0.14, 0.709 (0.04)
	TAU	56.15 (33.74) [78]	65.75 (34.68) [73]	70.29 (34.97) [70]	12.44, < 0.001 (0.58)	
SDQ—Total	B2S	14.61 (6.49) [61]	11.98 (6.82) [43]	10.22 (5.78) [41]	32.28, < 0.001 (1.55)	12.57, < 0.001 (0.69)
	TAU	14.52 (6.12) [58]	12.56 (5.73) [39]	12.81 (6.72) [37]	1.24, 0.271 (0.31)	
Emotional symptoms	B2S	5.57 (2.72) [61]	3.95 (2.97) [43]	3.51 (2.28) [41]	45.84, < 0.001 (1.79)	10.94, 0.001 (0.63)
	TAU	5.14 (2.60) [58]	4.15 (2.39) [39]	4.22 (2.86) [37]	3.53, 0.066 (0.52)	
Conduct problems	B2S	2.02 (1.81) [61]	1.63 (1.51) [43]	1.32 (1.51) [41]	5.82, 0.018 (0.50)	0.19, 0.662 (0.07)
	TAU	2.02 (1.73) [58]	1.59 (1.39) [39]	1.41 (1.59) [37]	6.16, 0.015 (0.56)	
Hyperactivity/Inattention	B2S	4.34 (2.73) [61]	4.09 (2.88) [43]	3.61 (2.31) [41]	12.65, < 0.001 (0.76)	2.34, 0.128 (0.28)
	TAU	4.90 (2.55) [58]	4.23 (2.28) [39]	4.30 (2.46) [37]	1.00, 0.319 (0.22)	
Problems with peers	B2S	2.67 (1.94) [61]	2.30 (1.87) [43]	1.78 (1.54) [41]	11.16, 0.001 (0.87)	13.69, < 0.001 (0.71)
	TAU	2.47 (1.94) [58]	2.59 (1.93) [39]	2.89 (2.18) [37]	4.20, 0.045 (0.57)	
Prosocial Behavior	B2S	7.61 (1.46) [61]	7.84 (2.09) [43]	8.20 (1.47) [41]	3.79, 0.054 (0.41)	0.64, 0.425 (0.12)
	TAU	7.26 (2.07) [58]	7.38 (1.84) [39]	7.38 (1.72) [37]	0.34, 0.559 (0.13)	
Impact	B2S	2.90 (3.02) [61]	2.21 (2.52) [43]	1.10 (1.62) [41]	14.94, < 0.001 (0.77)	6.94, 0.009 (0.39)
	TAU	2.69 (2.80) [58]	1.67 (1.83) [39]	2.59 (3.00) [37]	0.19, 0.661 (0.10)	
SDQ-P—Total	B2S	16.49 (5.52) [74]	12.85 (6.23) [65]	11.56 (6.32) [57]	62.04, < 0.001 (1.83)	9.11, 0.003 (0.49)
	TAU	15.19 (5.96) [78]	12.81 (5.49) [59]	12.45 (6.05) [53]	13.24, < 0.001 (0.85)	
Emotional symptoms	B2S	6.73 (2.50) [74]	4.80 (2.67) [65]	4.40 (2.28) [57]	72.22, < 0.001 (1.51)	4.18, 0.042 (0.26)
	TAU	6.32 (2.46) [78]	5.05 (2.53) [59]	4.53 (2.64) [53]	29.40, < 0.001 (0.98)	
Conduct problems	B2S	2.38 (1.89) [74]	1.77 (1.78) [65]	1.51 (1.72) [57]	26.71, < 0.001 (0.91)	6.12, 0.014 (0.32)
	TAU	1.95 (1.92) [78]	1.54 (1.59) [59]	1.53 (1.51) [53]	3.04, 0.084 (0.33)	
Hyperactivity/Inattention	B2S	4.27 (2.53) [74]	4.09 (2.47) [65]	3.37 (2.44) [57]	11.70, < 0.001 (0.60)	3.14, 0.078 (0.23)
	TAU	4.17 (2.49) [78]	3.80 (2.41) [59]	4.00 (2.67) [53]	0.93, 0.336 (0.18)	
Problems with peers	B2S	3.11 (2.06) [74]	2.18 (1.70) [65]	2.28 (1.92) [57]	15.82, < 0.001 (0.69)	4.03, 0.046 (0.26)
	TAU	2.76 (1.90) [78]	2.42 (1.73) [59]	2.40 (2.12) [53]	0.97, 0.328 (0.25)	
Prosocial Behavior	B2S	7.26 (2.28) [74]	7.45 (2.31) [65]	7.60 (2.12) [57]	1.90, 0.170 (0.25)	0.50, 0.480, (0.09)
	TAU	7.41 (1.98) [78]	7.47 (2.03) [59]	7.45 (2.01) [53]	0.22, 0.640 (0.09)	
Impact	B2S	4.93 (3.08) [74]	3.62 (3.30) [65]	2.88 (2.89) [57]	27.44, < 0.001 (1.21)	5.61, 0.019 (0.39)
	TAU	4.71 (2.92) [78]	3.86 (2.75) [58]	4.06 (3.37) [53]	3.74, 0.057 (0.46)	
SEQ-SS—Total	B2S	38.73 (9.41) [74]	43.19 (8.64) [53]	43.80 (9.08) [51]	36.44, < 0.001 (1.39)	7.63, 0.006 (0.46)
	TAU	39.94 (9.14) [78]	40.35 (9.02) [49]	40.79 (9.91) [48]	0.66, 0.420 (0.19)	
Academic/Social stress	B2S	18.81 (5.12) [74]	21.51 (4.45) [53]	21.92 (4.74) [51]	38.43, < 0.001 (1.17)	8.14, 0.005 (0.38)
	TAU	19.28 (4.99) [78]	19.82 (5.05) [49]	20.06 (6.13) [48]	2.53, 0.115 (0.30)	
Separation/Discipline stress	B2S	19.92 (5.75) [74]	21.68 (5.39) [53]	21.88 (5.67) [51]	16.96, < 0.001 (0.78)	4.88, 0.028 (0.29)
	TAU	20.65 (5.45) [78]	20.53 (5.01) [49]	20.73 (4.95) [48]	0.13, 0.715 (0.07)	
SEQ-RSAP	B2S	37.23 (6.26) [74]	41.77 (5.81) [65]	43.42 (6.23) [57]	58.54, < 0.001 (1.65)	11.53, < 0.001 (0.53)
	TAU	38.97 (5.62) [78]	40.79 (6.26) [58]	41.25 (5.56) [53]	8.10, 0.006 (0.65)	

*B2S *Back2School, *TAU *Treatment As Usual, *Pre *Pre assessment, *Post *Post assessment, *3FU *3 month follow-up

### Secondary Outcomes

#### Emotional, behavioral, and social difficulties

As shown in Table 3, there were significant time × group interactions related to difficulties as reported by youths on the SDQ, in favor of the B2S group. On the total scale, the B2S group improved by -4.39 points from 14.61 points and the TAU group improved by -1.71 points from 14.52 points (*F* = 12.57, *p* =  < 0.01,* d* = 0.69). On the emotional symptoms scale, the B2S group improved by -2.06 points from 5.57 points, and the TAU group improved by -0.92 points from 5.14 points (*F* = 10.94, *p* =  < 0.01,* d* = 0.63), On problems with peers scale, the B2S group improved by -0.89 points from 2.67 points, and the TAU group deteriorated by 0.41 points from 2.47 points (*F* = 13.69, *p* =  < 0.01,* d* = 0.71). On the impact scale, the B2S group improved by -1.80 points from 2.90 points, and the TAU group improved by -0.10 points from 2.69 points (*F* = 6.94, *p* =  < 0.01,* d* = 0.39).

For parent reports of youths’ difficulties as per the SDQ-P, there were significant time × group interactions, in favor of the B2S group. On the total scale, the B2S group improved by -4.93 points from 16.49 points, and the TAU group improved by -2.74 from 15.19 points (*F* = 9.11, *p* =  < 0.01,* d* = 0.49). On the emotional symptoms scale, the B2S group improved by -2.33 points from 6.73 points, and the TAU group improved by -1.79 points from 6.32 points (*F* = 4.18, *p* = 0.04,* d* = 0.26). On the conduct problems scale, the B2S group improved by -0.87 points from 2.38 points, and the TAU group improved by -0.45 points from 1.98 points (*F* = 6.12, *p* = 0.01,* d* = 0.32). On the impact scale, the B2S group improved by -2.05 points from 4.93 points, and the TAU group improved by -0.65 points from 4.71 points (*F* = 5.61, *p* = 0.02,* d* = 0.39). See Table 3 for effects of intervention groups over time.

#### Self-Efficacy

There were significant time × group interactions related to change in youths’ self-reported self-efficacy, in favor of the B2S group. On the total scale, the B2S group improved by 5.07 points from 38.73 points, and the TAU group improved by from 0.85 points (*F* = 7.63, *p* =  < 0.01,* d* = 0.46). On the academic/social stress scale, the B2S group improved by 3.11 points from 18.81 points, and the TAU group improved by 0.78 points from 19.28 points (*F* = 8.14, *p* =  < 0.01,* d* = 0.38). On the separation/discipline scale, the B2S group improved by 1.96 points from 19.92 points, and the TAU group improved by 0.08 points from 20.65 (*F* = 4.88, *p* = 0.03,* d* = 0.29). For parents’ reports of their own self-efficacy for responding to a SAP, a significant interaction was found, were the B2S group improved by 6.19 points from 37.23 points, and the TAU group improved by 2.28 points from 38.97 points (*F* = 11.53, *p* =  < 0.01,* d* = 0.53). See Table 3, for effects of interventions groups over time.

Explorative post hoc analyses were conducted for the time × group interactions. These analyses showed that the mean change difference was markedly higher in the time period from Pre to Post for all measures, with the exception of the youths reported SDQ-Impact where the mean change difference was highest from Post to FU (Online Resource 3).

### Adverse Effects and Treatment Satisfaction

The *ESQ* was completed by parents in 65 of the 74 families in the B2S group (88%), and 58 of the 78 families in the TAU group (74%). None of the parents in the B2S group reported that the intervention had caused their child to feel worse. In the TAU group, 3 of the 58 parents who completed the *ESQ* (5%) reported that the intervention had caused their child to feel worse. One of the 65 parents from the B2S group (1.5%) and 2 of the 58 parents from the TAU group (3.4%) reported feeling worse due to the received intervention. Parents in the B2S group rated their satisfaction significantly higher (*M* = 16.28, *SD* = 3.57) compared to parents in the TAU group (*M* = 9.50, *SD* = 4.92), *t*(103) = 8.65, *p* < 0.01, *d* = 1.58.

## Discussion

In this study, we compared outcomes of interventions for SAPs using two approaches: a manualized modular transdiagnostic CBT program called B2S, and TAU. Contrary to expectations, B2S did not yield a significant advantage in increasing school attendance over TAU. There were substantial within-group improvements in school attendance for both B2S (medium effect sizes: *d* = 0.73 for hours, *d* = 0.54 for days) and TAU (medium effect sizes: *d* = 0.60 for hours, *d* = 0.68 for days), but no significant differences between the two, refuting our first hypothesis. However, B2S outperformed TAU on most of the emotional, behavioral, and social difficulties measures. Effect sizes between the groups ranged from small to medium (*d* = 0.29–0.58) and included youth- and parent-reported ratings of total problems, emotional symptoms, and the impact of problems, youth-reported social difficulties related to peers, and parent-reported youths conduct problems, aligning with our second hypothesis. Additionally, B2S yielded significantly higher increases in youths’ self-efficacy (academic/social stress and separation/discipline stress) and parents’ self-efficacy. Effect sizes were significant for both youths (*d* = 0.29 and 0.47) and parents (*d* = 0.53), supporting our third hypothesis. Participants in the B2S group also reported significantly higher treatment satisfaction compared to TAU, with a large effect size.

The average intervention time for families in each condition was almost equal. The TAU group received a wide range of interventions from public and private service providers, averaging 13.4 h, closely matching the average 15.0 h for families in the manualized B2S intervention. Thus, the B2S intervention’s length was comparable to TAU, and it could probably be implemented in municipalities without extra costs except for B2S program training and supervision. While we lack specific details on TAU intervention content, Danish law mandates public schools to collaborate with parents to help youths attend school (Danish Ministry of Children and Education, 2019), likely resulting in many youths and families receiving school-initiated interventions to increase school attendance. Notably, 56 out of 60 youths in the TAU group received school-provided interventions (on average 11.0 h).

The TAU group demonstrated a notable increase in school attendance, surpassing the overall effect sizes reported in previous meta-analyses of interventions for truancy (*d* = 0.46) and school refusal (*d* = 0.54) (Maynard et al., 2013, 2018). This aligns with findings from Reissner and colleagues (2015), who also observed positive outcomes related to school attendance among participants receiving TAU (Reissner et al., 2015). Given the TAU group’s steadily declining attendance in the year preceding participation in the current study (Online Resource 2), it is likely that participation in TAU contributed to the increase in attendance. The increase in school attendance within the TAU group can be explained in several ways. First, it is possible that both the therapists providing B2S and the service providers in the TAU group were equally effective in improving school attendance. The superior outcomes in B2S, namely improvement in emotional, behavioral, and social difficulties, as well as increased self-efficacy for both youths and parents, and higher treatment satisfaction, may be attributed to the therapeutic components unique to B2S, including graded exposure and disorder-specific modules from the MMM manual. Second, there is a possibility of contamination, where participants in the control arm received active intervention (Magill et al., 2019). In our study, when families were randomly assigned to TAU, schools were informed that the family had been encouraged to contact them for help with their child’s SAP. This proactive approach may have led schools to intensify their support and parents to insist on more help from them. In total, 30 out of 44 schools (68%) had youths participating in both B2S and TAU, potentially influencing the support given to TAU families, because school personnel were familiar with B2S. Additionally, psychologists delivering B2S worked part-time on the project while also serving as school psychologists in municipalities during the study. Although they were instructed not to incorporate B2S elements in their work as school psychologist, their experience with B2S could have unintentionally influenced the intervention for those in TAU. Furthermore, schools may have adopted B2S school meeting practices in their work with TAU youths. This raises the possibility that elements from B2S inspired the intervention received by the TAU group, potentially enhancing its effectiveness. Third, a considerable portion of TAU participants received intervention from private providers, potentially impacting the TAU group’s outcomes. Specifically, nearly one-third of TAU youths (19 out of 60, or 32%) received on average 13.4 h of help from private providers, including private psychologists. Considering these findings, the substantial proportion of the participants in the TAU group receiving support from private providers, suggests that the available public services were not readily available in some instances, due to high demand or were insufficient to meet the needs of the youth and families.

Prior research has revealed discrepancies and weak associations in school attendance data reported by registries and parents (Keppens et al., 2019; Lomholt et al., 2020, Johnsen et al., 2022). This study addresses these issues by using both registry and parent-reported data as primary outcome measures. At baseline we found a large discrepancy between the attendance reported for the last 10 days in the school attendance registries (17.7% had 100% attendance) and what parents reported regarding their children's attendance (1.4% had 100% attendance). In the assessment of the sociodemographic characteristics and previous attendance patterns of the current sample (Johnsen et al., 2022), 22 parents reported that their child had 100% school absence in the three months before recruitment, while registry data showed only two participants had 100% school absence in the same time period (Johnsen et al., 2022). It is unlikely that nearly one-fifth of the youth participating in the current RCT trial had no absence at all from school at inclusion, since the families had sought treatment for SAP, and since the inclusion criteria was 10% absence or more. The results therefore question the reliability of the municipalities register data. Since it is mandatory for schools in Danmark to record absences rather than presence, we can speculate that schools might be omitting to register absences for children who have been absent for extended periods, automatically categorizing them as presents instead.

The study boasts several strengths, including the large sample of youths displaying SAPs, an ecologically valid design with close collaboration with municipalities and intervention conducted by their psychologists, and thus the use of a highly active comparator in TAU. However, there are noteworthy limitations. Firstly, potential contamination from the B2S condition may have influenced TAU effectiveness. Secondly, as the TAU group were free to seek and receive any help and support available (see Online Resource 1), we do not have information on the number of staff involved in the TAU intervention, nor do we know the level of training and expertise of the staff in the TAU intervention. This is a limitation, as the characteristics and training of TAU staff may have affected the outcomes of the TAU intervention. Additionally, we lack information on whether and to what extend B2S-trained staff may have provided intervention to TAU participants (although they were instructed not to). Lastly, concerns arise regarding the validity of the register-based measure of school attendance, as we uncovered discrepancies between parent-reported attendance and registry-based attendance data, with parent reports indicating lower attendance levels (Johnsen et al., 2022).

Our findings have clinical implications. The sample comprised youths with substantial school absence and emotional and behavioral symptoms at a clinical level, impacting their daily lives (Johnsen et al., 2022). B2S delivered in an outpatient, school-based setting yielded benefits in emotional, behavioral, and peer problems, overall life impact, and youths’ and parents’ self-efficacy. Thus, combined with high satisfaction rates reported by both youths and parents, suggest B2S as a viable intervention for SAPs in an outpatient, school-based setting. Notably, the psychologists conducting B2S were previously novices in CBT, but with brief training, manual use, and weekly supervision, they helped achieve positive outcomes in school attendance and mental health. These results, coupled with acceptable competence and adherence measures (Bjaastad et al., 2015) and significantly higher parent-rated satisfaction in the B2S group, suggest that non-expert clinicians can successfully administer B2S with proper training and supervision in an outpatient setting. Moreover, B2S outperforming TAU in reducing youths’ symptoms and enhancing their self-efficacy could have lasting positive effects on their learning and mental well-being. Strengthening self-efficacy may also serve as a protective factor in coping with future life challenges. These findings suggest, that B2S could increase the quality of treatment of SAPs in schools and other outpatient settings, possibly reducing barriers to care (Kearney & Benoit, 2022). Kearney and Graczyk (2014) have proposed a stepped care model featuring a three-tiered service delivery approach: universal, targeted, and intensive interventions for SAPs. Tier 1 interventions are directed toward all students; Tier 2 interventions are directed toward at-risk students who require additional support; Tier 3 interventions are directed toward students with severe or complex problems who require a more individualized and concentrated approach. Following the current evaluation, the B2S intervention seems applicable as an intervention for SAPs (e.g., truancy, school refusal, school withdrawal, and school exclusion; Heyne et al., 2019), in either Tier 2 or Tier 3. Nonetheless, B2S is not suitable for all youth with SAPs, as it requires motivation and commitment from the family to work on increasing school attendance.

To conclude, this study marks the first evaluation of a transdiagnostic CBT outpatient intervention for youths displaying SAPs using a rigorous experimental design. Contrary to our expectations, the B2S intervention did not significantly improve school attendance compared to TAU; both groups showed attendance improvements. However, as hypothesized, youths participating in B2S demonstrated greater improvement in emotional, behavioral, and peer problems, as well as self-efficacy, compared to TAU. Additionally, parents in the B2S group reported greater increase in self-efficacy in helping their child attend school regularly, compared to TAU. The next step in the B2S evaluation involves conducting an economic evaluation using pertinent data up to and including the school year of 2023/24. This evaluation will compare the B2S group with the TAU group, considering cost benefit assessed through obtained grades, youth education, employment, and income (Thastum et al., 2019). Future research should explore the enduring effects of B2S on school attendance and investigate whether changes in emotional, behavioral, and peer problems, and self-efficacy, mediate attendance outcomes. Further, given the non-significant difference between B2S and TAU with respect to school attendance, future research should investigate effect modification to identify subgroups that may benefit more from B2S based on factors such as school attendance levels, sociodemographic factors, family characteristics, the severity of youths’ emotional or behavioral problems, and psychiatric diagnosis (Heyne et al., 2015). Moreover, understanding what aspects of the B2S program work best for specific groups could inform intervention improvements.

## Supplementary Information

Below is the link to the electronic supplementary material.

Supplementary file1 (DOCX 24.8 KB)

## Data Availability

The data that form the basis of the manuscript submitted to you comprises information of both child and caregiver, and all participants were guaranteed full anonymity. As such and in line with Danish Data Protection Agency regulations, the data used in this study are not publicly available because they contain sensitive and potentially identifiable information. Unidentifiable data excerpts can be made available upon request to the corresponding author (mikael@psy.au.dk). Further data access requests may be sent to The Technology Transfer Office at Aarhus University (tto@au.dk).
