# Supplementary material for: The Effectiveness of Modular Transdiagnostic Cognitive Behavioral Therapy Versus Treatment as Usual for Youths Displaying School Attendance Problems: A Randomized Controlled Trial
Source: Res Child Adolesc Psychopathol. 2024 May 13;52(9):1397–412. doi: 10.1007/s10802-024-01196-8 (PMC11420258; doi:10.1007/s10802-024-01196-8)
Supplement: Supplementary file 1 — Supplementary file1 (DOCX 24.8 KB) [file 10802_2024_1196_MOESM1_ESM.docx]

| **Online Resource 1.** Descriptive information regarding the interventions received in the treatment as usual (TAU) group | | |
| --- | --- | --- |
|  | **TAU (*n* = 60)** | |
|  | ***n* (%)** | **Mean hours (SD)** |
| Received any intervention: | 60 (100) | 13.4 (21.6) |
| Number of service providers (i.e., school, municipal, region, or private): |  | - |
| *One provider* | 10 (16.7) | - |
| *Two different providers* | 23 (38.3) | - |
| *Three different providers* | 24 (40.0) | - |
| *Four different providers* | 3 (5.0) | - |
| Public services: | 59 (98.3) | 11 (22.0) |
| School services: | 56 (93.3) | 6.8 (18.6) |
| *School meeting* | 55 (91.7) | - |
| *Home schooling* | 8 (13.3) | - |
| *Special education* | 2 (3.3) | - |
| *Reduced school schedule* | 1 (1.7) | - |
| Municipal services: | 41 (68.3) | 6.4 (14.9) |
| *Meeting with social worker* | 21 (35.0) | - |
| *Interventions provided by school psychologist* | 13 (21.7) | - |
| *Interventions provided by clinical psychologist* | 7 (11.7) | - |
| *Mentoring program* | 7 (11.7) | - |
| *Meeting with an official from the municipality* | 5 (8.3) | - |
| *Enrollment or support from a youth center* | 5 (8.3) | - |
| *Multisystemic Therapy* | 2 (3.3) | - |
| Regional services: | 24 (40) | 3.3 (2.3) |
| *Psychiatric hospital (assessment or inpatient care)* | 16 (26.7) | - |
| *Hospital / MD practitioner* | 13 (21.7) | - |
| *Center for suicide prevention* | 1 (1.7) | - |
| Private services: | 19 (31.7) | 8.5 (8.4) |
| Private psychologist | 14 (21.7) | 5.8 (5.6) |
| Other private interventions: | 5 (8.3) | 8.5 (9.0) |
| *Physiotherapy* | 1 (1.7) | - |
| *Hypnotherapy* | 1 (1.7) | - |
| *Private tutoring* | 1 (1.7) | - |
| *Post adoption services* | 1 (1.7) | - |
| *Cancer survivor support* | 1 (1.7) | - |
| Note: The information is derived from a semi-structured interview conducted among parents in the TAU group, assessing the interventions received from pre to post. | | |

|  |
| --- |
| **Online Resource 2.** Mean monthly school attendance (%), for the 10 months prior to inclusion. |

| **Online Resource 3.** Sensitivity analysis of mean change and mean change difference with corresponding confidence intervals (95%) by group and by time period. | | | | | | |
| --- | --- | --- | --- | --- | --- | --- |
|  | Group | Pre-Post | | | Post-3FU | |
|  |  | Mean change (SD) [valid n] | M. diff. (95%CI) | Mean change (SD) [valid n] | | M. diff. (95%CI) |
| School Attendance - Hours, Two weeks (%) | B2S | 14.51 (31.23) [65] | 4.46  (-8.15, 17.08) | 12.94 (34.82) [52] | | -0.70  (-14.84, 13.44) |
|  | TAU | 10.05 (35.45) [46] |  | 13.64 (34.21) [43] | |  |
| School Attendance - Days, Two weeks (%) | B2S | 11.62 (37.09) [74] | 2.44  (-9.73, 14.62) | -1.53 (26.52) [72] | | -6.96  (-16.68, 2.77) |
|  | TAU | 9.18 (37.59) [73] |  | 5.43 (31.93) [70] | |  |
| SDQ - Total | B2S | 4.26 (5.26) [43] | 3.84  (1.69, 6.00) | 0.69 (4.87) [36] | | 0.24  (-1.93, 2.42) |
|  | TAU | 0.41 (4.46) [39] |  | 0.45 (3.90) [31] | |  |
| Emotional symptoms | B2S | 2.19 (2.60) [43] | 1.65  (0.53, 2.77) | 0.42 (2.02) [36] | | 0.26  (-0.64, 1.15) |
|  | TAU | 0.54 (2.49) [39] |  | 0.16 (1.57) [31] | |  |
| Problems with peers | B2S | 0.51 (1.88) [43] | 0.79  (0.03, 1.56) | 0.22 (1.29) [36] | | 0.42  (-0.32, 1.15) |
|  | TAU | -0.28 (1.57) [39] |  | -0.19 (1.70) [31] | |  |
| Impact | B2S | 1.21 (3.86) [43] | 0.57  (-0.85, 1.98) | 0.81 (2.20) [36] | | 1.39  (0.33, 2.45) |
|  | TAU | 0.64 (2.29) [39] |  | -0.58 (2.13) [31] | |  |
| SDQ-P - Total | B2S | 3.95 (4.86) [65] | 2.04  (0.38, 3.69) | 1.00 (4.22) [55] | | 0.91  (-0.78, 2.60) |
|  | TAU | 1.92 (4.41) [59] |  | 0.09 (4.31) [46] | |  |
| Emotional symptoms | B2S | 2.12 (2.70) [65] | 0.97  (0.08, 1.86) | 0.31 (1.93) [55] | | -0.23  (-1.04, 0.58) |
|  | TAU | 1.15 (2.27) [59] |  | 0.54 (2.17) [46] | |  |
| Conduct problems | B2S | 0.69 (1.38) [65] | 0.47  (0.00, 0.94) | 0.18 (1.11) [55] | | 0.10  (-0.35, 0.54) |
|  | TAU | 0.22 (1.23) [59] |  | 0.09 (1.13) [46] | |  |
| Problems with peers | B2S | 0.78 (1.68) [65] | 0.51  (-0.03, 1.06) | -0.02 (1.31) [55] | | 0.18  (-0.39, 0.75) |
|  | TAU | 0.27 (1.34) [59] |  | -0.20 (1.57) [46] | |  |
| Impact | B2S | 1.57 (3.11) [65] | 0.91  (-0.15, 1.98) | 0.45 (1.82) [55] | | 0.30  (-0.46, 1.06) |
|  | TAU | 0.66 (2.81) [58] |  | 0.16 (2.01) [45] | |  |
| SEQ-SS - Total | B2S | 5.68 (8.63) [53] | 5.11  (1.54, 8.68) | 0.45 (6.81) [44] | | -0.24  (-3.27, 2.80) |
|  | TAU | 0.57 (9.54) [49] |  | 0.69 (7.08) [39] | |  |
| Academic/Social stress | B2S | 3.36 (4.68) [53] | 2.73  (0.81, 4.64) | 0.39 (4.20) [44] | | -0.13  (-1.90, 1.64) |
|  | TAU | 0.63 (5.06) [49] |  | 0.51 (3.87) [39] | |  |
| Separation/Discipline stress | B2S | 2.32 (5.17) [53] | 2.38  (0.30, 4.46) | 0.07 (3.71) [44] | | -0.11  (-1.85, 1.63) |
|  | TAU | -0.06 (5.40) [49] |  | 0.18 (4.25) [39] | |  |
| SEQ-RSAP | B2S | 4.74 (6.40) [65] | 2.86  (0.71, 5.01) | 1.04 (4.73) [55] | | 0.46  (-1.48, 2.40) |
|  | TAU | 1.88 (5.55) [58] |  | 0.58 (5.02) [45] | |  |
| Note: B2S = Back2School, TAU = Treatment As Usual, Pre = Pre assessment, Post = Post assessment, 3FU = 3 month follow-up, 12FU = 3 month follow-up, SDQ = Strength and Difficulties Questionnaire, SDQ = Strength and Difficulties Questionnaire – Parent version, SEQ-SS = Self-Efficacy Questionnaire for School Situations, SEQ-RSAP = Self-efficacy Questionnaire for Responding to School Attendance Problems, M. diff (95%CI) = Mean change difference with 95% confidence difference. | | | | | | |
